# A platform of genetically engineered bacteria as vehicles for localized delivery of therapeutics: Toward applications for Crohn's disease

**DOI:** 10.1002/btm2.10113

**Published:** 2018-09-23

**Authors:** Ryan McKay, Monil Ghodasra, John Schardt, David Quan, Alex Eli Pottash, Wu Shang, Steven M. Jay, Gregory F. Payne, Matthew Wook Chang, John C. March, William E. Bentley

**Affiliations:** ^1^ Fischell Dept. of Bioengineering University of Maryland College Park MD; ^2^ Institute for Bioscience and Biotechnology Research University of Maryland College Park MD; ^3^ Women's Malignancies Branch, Center for Cancer Research, National Cancer Institute National Institutes of Health Bethesda MD; ^4^ Marlene and Stewart Greenebaum Comprehensive Cancer Center University of Maryland School of Medicine Baltimore MD; ^5^ Program in Molecular and Cellular Biology University of Maryland College Park MD; ^6^ Dept. of Biochemistry, Yong Loo Lin School of Medicine National University of Singapore Singapore; ^7^ NUS Synthetic Biology for Clinical and Technological Innovation, Life Sciences Institute National University of Singapore Singapore; ^8^ Dept. of Biological and Environmental Engineering Cornell University Ithaca NY

**Keywords:** biologic therapeutics, inflammatory bowel disease, motility, probiotics, protein secretion, signal amplification, synthetic biology, targeted delivery

## Abstract

For therapies targeting diseases of the gastrointestinal tract, we and others envision probiotic bacteria that synthesize and excrete biotherapeutics at disease sites. Toward this goal, we have engineered commensal E. coli that selectively synthesize and secrete a model biotherapeutic in the presence of nitric oxide (NO), an intestinal biomarker for Crohn's disease (CD). This is accomplished by co‐expressing the pore forming protein TolAIII with the biologic, granulocyte macrophage‐colony stimulating factor (GM‐CSF). We have additionally engineered these bacteria to accumulate at sites of elevated NO by engineering their motility circuits and controlling pseudotaxis. Importantly, because we have focused on *in vitro* test beds, motility and biotherapeutics production are spatiotemporally characterized. Together, the targeted recognition, synthesis, and biomolecule delivery comprises a “smart” probiotics platform that may have utility in the treatment of CD. Further, this platform could be modified to accommodate other pursuits by swapping the promoter and therapeutic gene to reflect other disease biomarkers and treatments, respectively.

## 1 INTRODUCTION

Inflammatory Bowel Disease (IBD) is a term that describes a variety of ailments, most commonly Ulcerative Colitis (UC) or Crohn's disease (CD). The prevalence of these diseases is on the rise globally, with an estimated 2 million new cases across North America and Europe over the last decade.^1^, ^2^ The largest category of therapies for IBD, biologics, are generally the most effective, but entail invasive injections and consequently systemic side effects.^3^, ^4^, ^5^, ^6^, ^7^ Given the ability to treat IBD with biologics and its localization to the gastrointestinal (GI) tract, it is possible to utilize bacteria as vehicles for both the synthesis and delivery of a therapy, referred to as “smart” or “turbo” probiotics.^8^, ^9^, ^10^, ^11^, ^12^, ^13^, ^14^, ^15^, ^16^ The majority of such applications solely investigate the production of a biotherapeutic, yet few have addressed targeting mechanisms for localized delivery. We suggest this may be accomplished via a phenomenon known as pseudotaxis, whereby a cell's motility is regulated by a chemical to result in an accumulation of cells within a gradient of the signal; this targeting mechanism has been documented in bacteria with the goals of “smart probiotics” in mind.^17^, ^18^, ^19^, ^20^, ^21^

Briefly, in this work pseudotaxis is achieved via the production of a motility regulator protein CheZ with a C‐terminal degradation tag, YbaQ, in a *cheZ*‐null mutant (Δ*cheZ*).^18^, ^21^ Cells lacking CheZ are unable to swim forward and they tumble continuously,^22^, ^23^ and when a chemical cue induces expression of CheZ‐YbaQ, motility is restored and cells generally accumulate near sources of the chemical, referred to as a pseudoattractant.^21^ Using a microfluidic device with controlled gradients,^24^ we have demonstrated controlled pseudotaxis in response to a signaling molecule of the opportunistic pathogen, *Pseudomonas aeruginosa.* 21 In this work, motility was characterized via the run:tumble ratio of cells, and similarly their overall speed, as more runs correspond with faster cells. In order to achieve pseudotaxis in a CD‐relevant translational setting, it is important to have a disease‐specific chemical cue at the desired site (i.e., a biomarker) and it must be able to elicit a genetic response from the engineered bacteria. In patients with IBD, the chemical nitric oxide (NO) is a biomarker found in the intestinal fluid at levels approximately 100‐fold higher than in healthy patients,^25^, ^26^ generated by intestinal epithelial cells that have been stimulated by pro‐inflammatory cytokines produced by nearby T‐cells.^25^, ^27^, ^28^, ^29^, ^30^, ^31^ Nitric oxide is often produced by macrophages as an antimicrobial,^32^, ^33^, ^34^ and various bacteria have evolved a natural genetically actuated defense mechanism for survival. In this work, we tap into this natural signal transduction mechanism using an NO‐responsive promoter to produce CheZ‐YbaQ and confer pseudotaxis.^35^, ^36^, ^37^ In addition, we can analogously regulate expression of a biotherapeutic to treat disease, such as Crohn's disease.

In the context of a “smart” probiotic, the free radical NO is an ideal signaling molecule as its presence is localized due to its short half‐life. Thus, engineered commensal bacteria should remain in an “off” state until they encounter the appropriate dose of NO, which would most likely occur at or near the site where a therapeutic is desired. Importantly, in our strategy, significant effort is made to minimally alter the engineered probiotics so that when they are in the “off” state, they function as they would normally function in native microenvironments. In recent articles, including from our group, with few exceptions,^8^, ^18^, ^38^, ^39^, ^40^ efforts to engineer motility or an ability to target have been lacking.^15^, ^41^, ^42^, ^43^, ^44^ In the present study, we have systematically examined the ability of engineered *E. coli* to respond to the NO cue and exhibit motility behaviors that suggest pseudotaxis toward NO, as indicated by observations parallel to those in our previous demonstration of pseudotaxis.^21^

That is, we had previously shown that when (a) CheZ deficient cells were restored to swim at speeds near the wild‐type controls (~15 μm/s) due to induced CheZ‐YbaQ production, and (b) accompanying run:tumble ratios were similarly restored to the WT levels, and (c) when swimming was slowed due to CheZ degradation (via inclusion of a degradation tag), pseudotaxis was achieved. These design studies were validated using controlled chemical gradients within a microfluidic device.^21^ Accordingly, they enabled subsequent de novo evaluation of alternative genetic circuits based on the engineered cells meeting these established motility metrics. This reduces dependence on complicated *in vitro* or *in vivo* analyses that involve signal cues that are unstable or where geometries are less defined. For example, *in vitro* studies of NO rely on its release from NONOates, which in turn, rely on pH gradients for NO to dissociate from the parent compound. As a consequence, NO gradients are superimposed onto pH gradients and the former is a relatively unstable molecule, making it impractical to evaluate NO‐targeted pseudotaxis within microfluidics. Instead, via the observation of metrics such as swimming speed with regards to the guidelines established for pseudotaxis from our earlier work,^21^ we can characterize pseudotactic behavior without complex microfluidic assays.

The model biotherapeutic used in this study is granulocyte macrophage‐colony stimulating factor (GM‐CSF). This protein is chosen based on its reported therapeutic effects for individuals with CD,^45^, ^46^, ^47^, ^48^ and proven production in bacterial hosts.^49^, ^50^, ^51^, ^52^ Briefly, CD is believed to have numerous contributing causes, comprising of a dysfunctional innate immune system (i.e., neutrophils) and a compromised mucosal barrier in the intestines.^5^, ^45^, ^46^, ^48^, ^53^, ^54^ Additionally, pathogenic bacteria can penetrate the intestinal mucosa and induce a strong inflammatory response, which would otherwise be mitigated by functioning neutrophils. GM‐CSF is reported to address each of these root causes, with capabilities to restore mucosal barrier functions via interactions with receptors on epithelial cells, a well‐characterized ability to stimulate neutrophils, and a recently discovered ability to be an agonist against pathogenic bacteria.^5^, ^46^, ^55^, ^56^, ^57^, ^58^ Thus, it has been investigated in clinical trials as a potential therapy, albeit via intravenous (i.v.) delivery.^45^, ^46^, ^47^ In a trial conducted by Braat et al., whereby probiotics were used to deliver cytokines to treat CD, the authors suggest localized delivery may increase efficacy versus systemic administration, and thus may also promote GM‐CSF's purported mucosal healing benefits.^5^, ^15^, ^46^, ^59^

In addition to engineering host motility, we created strains that express GM‐CSF with an N‐terminal *ompA* sequence for translocation into the periplasm, an environment often necessary for proper folding of recombinant proteins.^60^, ^61^, ^62^ Expression of recombinant GM‐CSF is also regulated by nitric oxide, but expression levels are amplified via a dual‐plasmid system utilizing the *T7lac* promoter that expresses T7 Polymerase (T7Pol) based on NO and T7Pol then amplifies the overall GM‐CSF synthesized. We have engineered this regulatory genetic circuit to minimize the off‐target effects such as the associated metabolic burden.^37^, ^63^

To assist in GM‐CSF export, the pore‐forming protein TolAIII is co‐expressed in the engineered cells with a signal sequence that guides it to the outer membrane.^64^, ^65^ Taken together, GM‐CSF is first shuttled into the periplasm, and subsequently released into the extracellular space via the pores formed by TolAIII. The incorporation of a release mechanism for GM‐CSF is important, as a biotherapeutic that remains intracellular is less likely to perform its desired function outside of the bacterial vehicle. Such approaches that have appeared thus far include inducing host cell lysis via colicin proteins,^8^ or the synthesis of fusion proteins comprising the active therapeutic and a carrier such as the YebF protein.^18^, ^66^, ^67^ We note that induced lysis requires finely tuned initiation of cell death: premature lysis results in insignificant accumulation of the biotherapeutic and delayed lysis could release the recombinant protein too late. Both scenarios could diminish the efficacy of the therapy‐producing bacteria. Here, we opted to form pores in the outer membrane with goals being to: (a) increase the flux of periplasmic GM‐CSF out of the cells (eliminating the YebF shuttling mechanism), and (b) ensure biologic activity and avoid unintended side effects by using a minimally modified GM‐CSF protein.

Using these designed bacteria, the native promoter (*hmp*) facilitates the sensing of NO to regulate the synthesis and release of GM‐CSF, as well as guide the cells to the desired sites via pseudotaxis. Our hypothesis is that through selective induction to produce the therapeutic at NO‐rich locales (sites of inflammation), we may reduce systemic side effects and increase efficacy of the biopharmaceutical GM‐CSF. As probiotics are designated safe for ingestion and occasionally even administered to patients with IBD,^3^, ^68^, ^69^, ^70^, ^71^, ^72^, ^73^ we believe that the methodologies developed here provide robust preclinical designs for uniquely delivering biologics in the GI tract. That is, the experimental platform or “test track” efforts described in this work, in conjunction with our previous design studies,^21^ enable engineering assessment of the parameters needed for future animal studies and ultimately, human use.

## 2 MATERIALS AND METHODS

### 2.1 Plasmid construction

Details are presented in Supporting Information. Oligonucleotides are available upon request, and a list of plasmids used is presented in Table 1.

**Table 1:** List of plasmids used

Plasmid	Characteristics	Reference
pFZYl	Promoterless galK‐lacZYA transcriptional fusion vector, Ap^r^	Koop et al.^74^
pT5G	pET200 derivative, containing egfp under the T5 phage promoter, Km^r^	Tschirhart et al.^75^
pTA3Y	pACYC177 derivative encoding a gene fusion between the ribose binding protein signal sequence and TolAIII, Km^r^	Wan and Baneyx^65^
pXYZ202	Expression vector containing cheY** (cheY^13DK106YW^), Apr	Zhu et al.^76^
pGM29ompA	Expression vector containing ompA‐gmcsf with a C‐terminal c‐myc/His6 tag, Ap^r^	Sletta et al.^50^
pBbS8a	Low copy cloning vector, Ap^r^	Lee et al.^77^
pBSh	pBbS8a shortened: pBbS8a lacking the pBAD promoter, Ap^r^	McKay et al.^37^
pET200	Cloning vector, containing T7lac promoter, Km^r^	Invitrogen
pST39	Cloning vector with restriction sites to facilitate polycistronic expression, Ap^r^	Song Tan^78^
pWM1	pFZY 1 derivative, containing cheZ with the C‐terminal YbaQ tag, under the soxRS promoter region, Ap^r^	McKay et al.^21^
pHW01	pFZY 1 derivative, containing cheZ under the soxRS promoter region, Ap^r^	Tschirhart et al.^75^
pRM11	pBSh derivative, containing cheZ under the hmp promoter, Ap^r^	This study
pRM24	pBSh derivative, containing cheZ with the C‐terminal YbaQ tag under the hmp promoter	This study
pRM31	pBSh derivative, containing cheY under the hmp promoter, Ap^r^	This study
pRM32	pBSh derivative, containing cheY** under the hmp promoter, Ap^r^	This study
pRM44	pBSh derivative, containing T7Pol under the hmp promoter with 16 inserted bases before the start codon, Ap^r^	McKay et al.^37^
pRM45	pBSh derivative, containing T7Pol under the hmp promoter with 16 inserted bases before the start codon and cheZ with the C‐terminal YbaQ tag expressed polycistronically, Ap^r^	This study
pRM52	pBSh derivative, containing T7Pol under the hmp promoter with six extra bases before the start codon and lysY expressed polycistronically, Ap^r^	McKay et al.^37^
pRM60	pBSh derivative, containing ompA‐gmcsf‐c‐myc‐his6 under the hmp promoter, Ap^r^	This study
pRM101	pET200 derivative, containing ompA‐gmcsf‐c‐myc‐his6 under the T7lac promoter, Km^r^	This study
pRM102	pET200 derivative, containing ompA‐gmcsf‐c‐myc‐his6 and rbp‐tolAIII expressed polycistronically under the T7lac promoter, Km^r^	This study

### 2.2 Strains and growth conditions

K‐12 W3110 or Nissle 1917 *E. coli* (referred to as Nissle in this study) are used for all experiments for which data are presented. TOP10 *E. coli* cells (Invitrogen, Carlsbad, CA) are used for subcloning. Cells are grown overnight and re‐inoculated the following day in LB media supplemented with appropriate antibiotics at 50 mg/ml. For experiments, cells are spun down at 2,000 g for 10 min near an OD_600_ of 0.2 and resuspended in M9 minimal media supplemented with glucose (0.4% wt/vol), vitamin B1 (20 μg/ml), and Casamino acids (0.5 g/L) with appropriate antibiotics to an OD_600_ of 0.2. Following, cells are induced with DPTA/NONOate (Cayman Chemical, Ann Arbor, MI) at 50 μM, unless otherwise specified, as desired. Cells are grown at 37 °C, shaking at 250 rpm. A list of all strains is found in Table 2.

**Table 2:** List of bacterial strains used

Strain	Relevant genotype	Reference
W3110 (WT)	K‐12 wild type, γ ‐, F^−^, IN(*rrnD‐rrnE*)1, rph‐1s	Genetic Stock Center Yale University, New Haven, CT
Δ*cheZ*	W3110 Δ*cheZ*	Tschirhart et al.^75^
WM10	W3110 WT with pFZY1	McKay et al.^21^
WM11	W3110 WT with pFZY1 and pT5G	McKay et al.^21^
WM12	W3110 Δ*cheZ* with pFZY1 and pT5G	McKay et al.^21^
WM13	W3110 Δ*cheZ* with pFZY1	McKay et al.^21^
RM07	W3110 Δ*cheZ*Δ*hmp*::Cm^r^	This study
RM11	W3110 Δ*cheZ* with pRM11	This study
RM12	W3110 Δ*cheZ*Δ*hmp*::Cm^r^ with pRM11	This study
RM15	W3110 Δ*hmp*::Cm^r^ with pT5G	This study
RM16	W3110 Δ*cheZ*Δ*hmp*::Cm^r^ with pT5G	This study
RM21	W3110 Δ*cheZ* with pRM11 and pT5G	This study
RM22	W3110 Δ*cheZ*Δ*hmp*::Cm^r^ with pRM11 and pT5G	This study
RM24	W3110 Δ*cheZ* with pRM24 and pT5G	This study
RM31	W3110 WT with pRM31 and pT5G	This study
RM32	W3110 WT with pRM32 and pT5G	This study
RM44	W3110 WT with pRM44 and pRM100	McKay et al.^37^
RM60	W3110 WT with pRM60	This study
RM74	W3110 WT with pRM44 and pRM101	This study
RM75	W3110 WT with pRM44 and pRM102	This study
RM80	W3110 WT with pRM52 and pRM101	This study
RM84	W3110 Δ*cheZ* with pRM45 and pRM101	This study
RM85	W3110 Δ*cheZ* with pRM45 and pRM102	This study
RM94	Nissle with pRM44 and pRM101	This study
RM95	Nissle with pRM44 and pRM102	This study

### 2.3 Cell lysate preparation

For Western Blotting: Cells were grown and resuspended in supplemented M9 minimal media as described above and induced as desired. Samples (~5 ml volume) were spun down at chosen timepoints at 10,000 g for 10 min. The supernatant was discarded, and the remaining pellets were flash frozen in liquid nitrogen and stored at −80 °C. Upon thawing, samples were lysed in 200 μl BugBuster HT (Novagen, Madison, WI) supplemented with 2.0 μl protease inhibitor cocktail, and spun down according to the manufacturer's protocol. Lysate concentrations were assessed via BCA assay (Pierce, Rockford, IL) and were normalized to the lowest sample's concentration (generally ~700 ng/μl) and boiled with SDS loading dye. For protein quantification: Cells were grown and resuspended in supplemented M9 media to an OD_600_ of 0.2 as described above and induced as desired. Cell pellets were derived from 15 ml culture and resuspended in 1 ml BugBuster HT with 10 μl protease inhibitor cocktail. An aliquot is removed for analysis via BCA assay, and the remainder is diluted to 15 ml with M9 media supplemented as above minus antibiotics. The solution is then concentrated using Amicon‐15 spin‐filters (EMD Millipore, Burlington, MA) with a 3,000 MWCO. Samples are spun at 3,220 g in a swinging bucket rotor for 1 hr at 16 °C. Concentrates are normalized to 450 μl and supplemented with 4.5 μl protease inhibitor cocktail and frozen at −80 °C.

### 2.4 Supernatant preparation

Cells were grown and resuspended in supplemented M9 media to an OD_600_ of 0.2 as described above and induced as desired. After 3 hr of induction, cells are pelleted and 15 ml supernatant is filtered with a 0.2 μm syringe filter. The solution is then concentrated using Amicon‐15 spin‐filters with a 3,000 MWCO. Samples are spun at 3,220 g in a swinging bucket rotor for 1 hr at 16 °C. Concentrates are normalized to 450 μl and supplemented with 4.5 μl protease inhibitor cocktail. An aliquot is removed and boiled with SDS loading dye as desired. Supernatants were stored at −80 °C.

### 2.5 Western blotting

Approximately 13.5 μg or 20 μg total protein per sample (for 15 or 10 well gel, respectively) was loaded in a 12.5% SDS‐PAGE gel and transferred to a nitrocellulose membrane using the semi‐dry Trans‐Blot SD cell (Bio‐Rad, Hercules, CA). Blots were blocked overnight at 4 °C with Tris‐buffered saline with 0.1% Tween‐20 (TBST) and 10% nonfat milk. Blots against the CheZ protein are described previously.^21^

### 2.6 Motility videos and analysis

Cells were grown and resuspended in supplemented M9 media to an OD_600_ of 0.2 as described above and induced as desired. At the desired time points, a small volume of cells (200 μl) are spun down slowly (400 g) for 5 min, and resuspended in an approximately 40–80 μl volume of chemotaxis buffer (CB; 1× PBS, 0.1 mM EDTA, 0.01 mM l‐methionine, 10 mM d,l‐lactate). Videos of fluorescent cells are recorded and analyzed as described previously.^21^, ^79^ For videos of cells using brightfield microscopy, cells were analyzed using a modified version of TumbleScore. Briefly, each pixel of the grayscale matrix was first subtracted from 255. Following, each image was subject to a range filter and a 2‐D order‐statistic filter. Finally, the images were segmented using Otsu's method of thresholding as in the unmodified TumbleScore to allow for subsequent analysis without further coding changes.^80^

### 2.7 Protein quantification

A competitive enzyme linked immunosorbent assay (ELISA) was performed using a His‐tag detection kit, following manufacturer's instructions (Genscript, Piscataway, NJ). Dilutions were first optimized with a trial assay as per the manufacturer's recommendation. Generally, a 100‐fold dilution of samples was the optimal dilution used in the assay. Plotted values are corrected based on the concentration factor (33.3‐fold using the Amicon spin‐filter) and normalized between samples using the total cellular protein as determined by the BCA assay.

### 2.8 Mammalian cell culture

The TF‐1 cell line was obtained from Dr David Stroncek (National Cancer Institute, Bethesda, MD). TF‐1 cells were maintained in RPMI 1640 (ATCC, Manassas, VA) with 10% FBS, 1% Pen‐Strep, and 2 ng/ml recombinant human GM‐CSF (Peprotech, Rocky Hill, NJ). For Caco‐2 studies, cells are handled as described previously.^37^ Briefly, Caco‐2 cells (ATCC, Manassas, VA) are seeded at 3 × 10^5^ cells/cm^2^ on rat collagen type I (Corning, Corning, NY) coated 24‐well transwell inserts. Cells are cultured in DMEM with 20 mM HEPES, 1% non‐essential amino acids, 1% Pen‐strep, 4.5 g/L d‐glucose, 4 mM l‐glutamine, 110 mg/L sodium pyruvate with 10% fetal bovine serum at 37 °C with 5% CO_2_. Cells were maintained for 24 days to ensure post‐confluent differentiation. Transepithelial electric resistance (TEER) was measured weekly to verify confluence. On Day 24, media without antibiotics but including 200 nM phorbol 12‐myristate 13‐acetate (PMA) and 10,000 U/ml interferon‐gamma (IFN‐γ) is added to the basolateral side of the cells. Media without antibiotics is added to the apical side, and incubated for 24 hr. Approximately 3 × 10^7^ log‐phase bacteria in PBS are added to the apical side after the 24 hr, and incubated for 90 min. The apical volume is then removed and bacteria are pelleted and RNA is extracted as described below.

### 2.9 Cellular proliferation assay (GM‐CSF biologic activity)

TF‐1 cells were seeded at 20,000 cells per well, in 250 μl media supplemented with 10% FBS, 1% Pen‐Strep, and in a 24 well plate, treated with 250 μl of the indicated factor or control, incubated in 5% CO_2_ at 37 °C for 4 days, and analyzed using Alamar Blue (Bio‐Rad, Hercules, CA) following the manufacturer's protocol. Supernatant factors are first diluted 16.6‐fold in supplemented media prior to further dilution for the assay to 250 μl (i.e., 10 μl of 16.6‐fold diluted cellular supernatant with 240 μl media). Controls are prepared in the same fashion (i.e., 10 μl of 16.6‐fold diluted lysate or M9 media with 240 μl media).

### 2.10 Quantitative PCR (qPCR)

Cells were grown and resuspended in supplemented M9 media to an OD_600_ of 0.2 as described above and induced as desired. At each timepoint, approximately 2 × 10^8^ cells are spun down, supernatant is removed, and the pellet is flash frozen in liquid nitrogen and subsequently stored at −80 °C. RNA extraction was performed using the TRIzol Max Bacterial RNA Isolation Kit (Invitrogen, Carlsbad, CA). Extracted RNA was normalized to approximately 25 ng/μl and treated with DNase I (Sigma, St. Louis, MO). qPCR was performed using SensiFAST SYBR Hi‐ROX One‐Step Kit (Bioline, Taunton, MA) with approximately 20 ng of total RNA per reaction. Each reaction was performed in triplicate, with outlying data removed resulting in some RNA samples only having a duplicate data point. 16s rRNA was used as the endogenous housekeeping gene. Data was analyzed using the ΔΔCt method as described in a published guide by Applied Biosystems, with the Ct threshold set automatically by the Applied Biosystems 7300/7500 SDS software for all samples. For qPCR studies on bacteria exposed to Caco‐2 cells, isolated RNA was normalized to 2.5 ng/μl with 2 ng total RNA per reaction.

## 3 RESULTS AND DISCUSSION

### 3.1 Generating a nitric oxide regulated motility circuit

**Figure 1:**
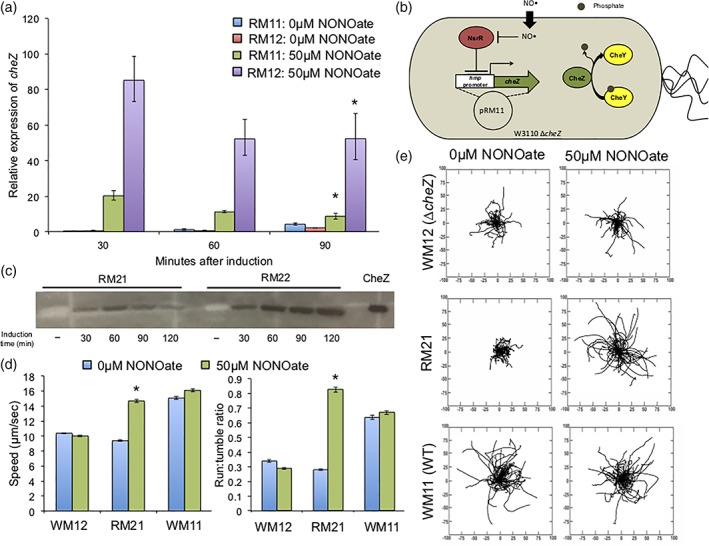
(a) Quantitative PCR on cheZ mRNA, using 16srRNA as a housekeeping gene. Samples are performed in triplicate and normalized as a fold‐change against RM11 cells at the zero timepoint (time of induction). Error bars represent the expression levels within one standard deviation, (b) schematic of an RM11 cell: a ΔcheZ host bacterium with plasmid pRM11. (c) Western blot against CheZ in cells induced with 50μM NONOate. (−) Indicates cells without induction grown for 90 min in M9 media. Equivalent total protein is added per well as determined via the BCA assay, (d) motility data: speed and run‐to‐tumble ratios determined using TumbleScore (Pottash et al. 2017). Cells are grown with or without induction for 90 min. Error bars are standard error, (e) rose graphs derived from data points in (d). Distance on axes is μm. * Indicates *p* < .05 by ANOVA, to compare the induced sample to its un‐induced counterpart

Our strategy in this work is to use non‐motile bacteria as the chassis and induce the motility upon recognition of the appropriate cue, promoting directional swimming. This is followed by “braking” in the absence of the cue owing to the inclusion of a degradation tag on the motility protein, CheZ. That is, in order to induce and restore motility, non‐motile Δ*cheZ* cells are required as a template as discussed earlier. Cells additionally lacking *hmp* (Δ*cheZ*Δ*hmp*) were generated with the goal of increased NO‐induced expression of *cheZ.* The Hmp protein removes NO by converting it to nitrate, thus Δ*hmp* cells yield a higher response when activated with NO due to prolonged NO exposure.^37^, ^81^ Removing *hmp* from the genome does not have an inherent effect on motility, demonstrated in Supporting Information Figure S1. Thus, RM11 (Δ*cheZ* host) and RM12 (Δ*cheZ*Δ*hmp* host) cells were developed, which produce CheZ in response to NO under the *hmp* promoter (Figure 1b). In Figure 1a, it is readily seen that cells induced with 50 μM NONOate (a category of molecules which release NO at physiological pH) exhibit increased expression of *cheZ* versus uninduced control cells. We note that in the absence of NONOate there remained a background level of expression. Importantly, cells lacking *hmp* produced at least fourfold more *cheZ* mRNA than *hmp* ^+^ bacteria (WT). These findings are corroborated in Figure 1c which shows the CheZ protein levels within these cells. In Figure 1c–e, RM21 and RM22 cells are RM11 or RM12 cells respectively, that were transformed with the pT5G plasmid to confer fluorescence (eGFP production) for imaging purposes. The effect of pT5G on CheZ production was negligible (Supporting Information Figure S1c). Importantly, uninduced cells showed no detectable CheZ on the Western blots. We hypothesized that while CheZ may be present, its effect should be minimal. Also, the decrease in band intensity seen in RM21 cells over time is likely attributed to the quick depletion of NO in the cells and media due to the action of Hmp, as noted previously.^81^, ^82^ Figure 1d provides a proof of concept that RM21 cells have a similar speed and run:tumble ratio to Δ*cheZ* cells when uninduced, and to wild‐type cells when induced for 90 min. Rose plots (Figure 1e) are shown to visualize the motility behavior of these cells. RM22 cells in the presence of NO, while producing CheZ, were completely immobile. Plasmid‐free Δ*hmp* cells were treated with NO and observed under the microscope and were also immobile, and thus the nitrosative stress of NO on cells lacking *hmp* completely abolishes their ability to move. Videos of Δ*hmp* samples are provided online in the Supporting Information.

**Figure 2:**
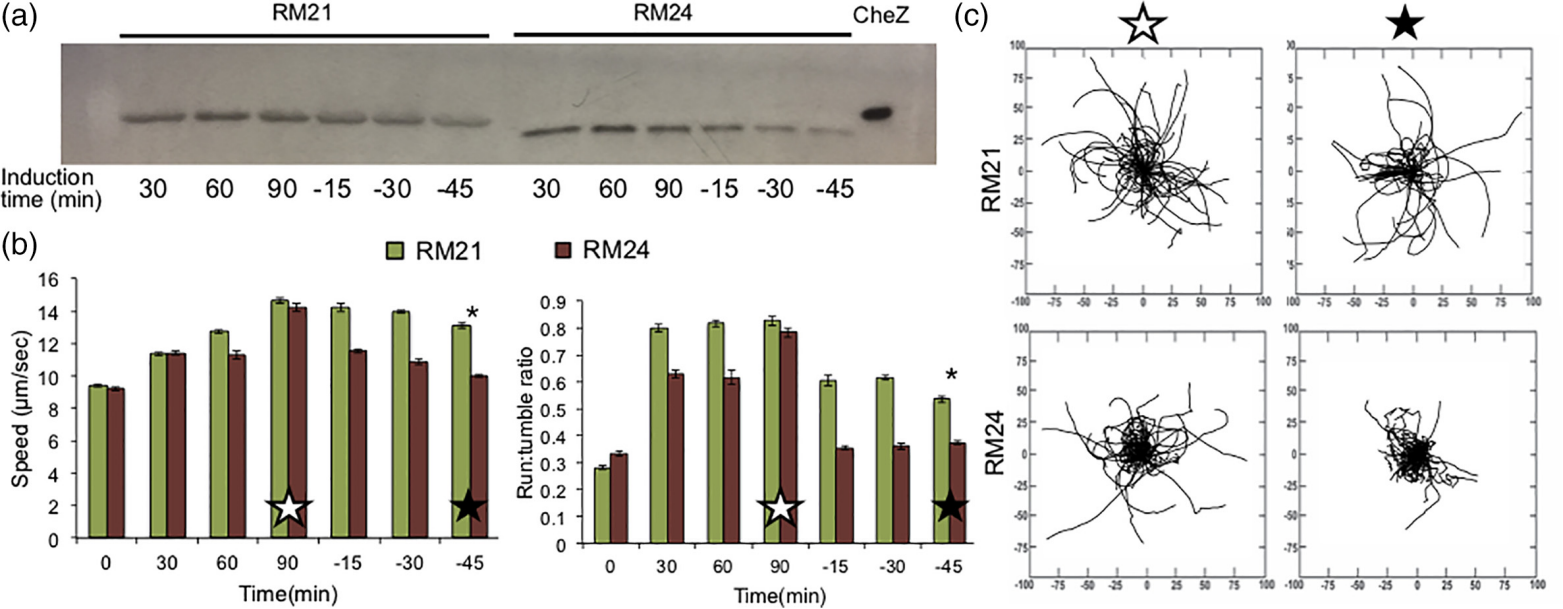
(a) Western blot against CheZ. Cells are induced with 50 μM NONOate for 90 min, then resuspended in fresh M9 media with no inducer and grown for 45 min. Equivalent total protein is loaded per well as determined via the BCA assay, (b) motility data: speed and run‐to‐tumble ratios determined using TumbleScore of cells described in (a). Error bars are standard error, (c) rose graphs derived from data points in (b). Open star refers to cells induced for 90 min. Closed star refers to cells induced for 90 min, then resuspended in fresh M9 media with no inducer and grown for 45 min. Distance on axes is μm. * Indicates *p* < .05 by ANOVA, to compare the RM24 sample to RM21 at the final time‐point

To achieve pseudotaxis, the inclusion of a proteolytic degradation tag on CheZ was included, as previously documented.^21^ RM24 cells are RM21 cells where CheZ is produced with the YbaQ degradation tag engineered into its C‐terminus. Figure 2a presents a comparison of these cells' CheZ levels during a course of induction and subsequently upon removal of inducer (NO) from the media. A noticeable loss of CheZ was observed in RM24 cells compared to RM21 cells after the 90 min induction period. The motility phenotype of the cells in Figure 2b is rather indicative: the speeds and run:tumble ratios of RM24 cells decreased more quickly and more dramatically than RM21 cells. The rose plots in Figure 2c help to visualize that RM24 cells 45 min after a 90 min induction are significantly less motile than RM21 cells in the same conditions.

**Figure 3:**
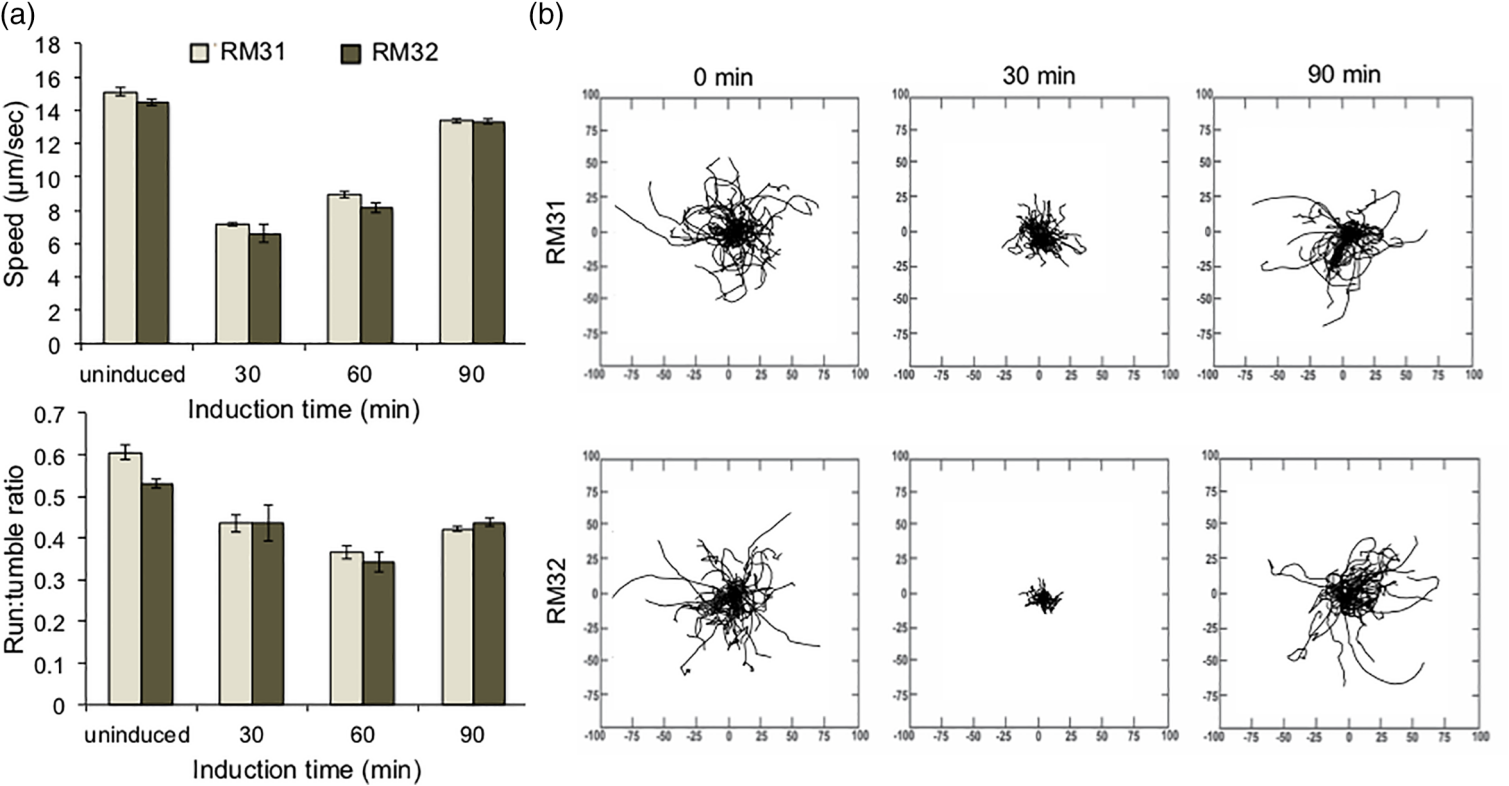
(a) Motility data: speed and run‐to‐tumble ratios determined using TumbleScore on cells overproducing CheY or Chey**, induced with 50μM NONOate. Error bars are standard error, (b) rose graphs derived from data points in (a). Distance on axes is μm

While pseudotaxis or guided motility often relies on expression of the “accelerator” CheZ in Δ*cheZ* cells, Tien et al. have looked at production of the protein CheY in WT cells to act as a “brake” in a desired area.^83^ Briefly, CheY promotes tumbling and when dephosphorylated by CheZ, cells will move forward or “run.”^84^, ^85^ Thus, a high CheY:CheZ ratio should encourage tumbling and so if cells are induced to overexpress CheY, they should remain in the vicinity of the inducer as a consequence of poor motility, or frequent tumbling. To assess this strategy versus CheZ‐mediated pseudotaxis, we synthesized RM31 and RM32 cells, expressing CheY or a mutant CheY (CheY**) that should be impervious to dephosphorylation by CheZ and thus produce a strong tumbling phenotype, in response to NO via the *hmp* promoter.^76^, ^86^ Figure 3 presents the results of this approach, ultimately indicating that with our NO‐mediated production of CheY or CheY**, pseudotaxis will not be achievable. While both RM31 and RM32 quickly become less motile upon induction, over the course of 90 min a full restoration of motility was observed. Likely, the generation of genomic CheZ was eventually able to balance the increase in its substrate, CheY, after induced. Surprisingly, no difference was observed between RM31 and RM32 cells, and thus CheY** may be able to be dephosphorylated by CheZ despite previous reports. However, it is also possible that expression of CheY (or CheY**) slows and subsequently protein levels drop over time, noting the deterioration of expression under the *hmp* promoter as observed in Figure 1.

### 3.2 Generating cells to secrete GM‐CSF, regulated by nitric oxide

With the goal of overproducing GM‐CSF under the *hmp* promoter, an initial concept would be to utilize the high‐production Δ*hmp* cells. However, as discussed above, cells lacking genomic *hmp* are immobile and thus the notion of a bacterium that can overproduce GM‐CSF and at the same time migrate toward NO sources is a significant challenge based on the use of the same promoter. Fortunately, a dual‐plasmid system employing the *T7lac* promoter to amplify a response to nitric oxide has been developed by our group.^37^ Using this genetic circuit, the T7 RNA Polymerase is generated under the *hmp* promoter on one plasmid (the relay plasmid), which leads to significant expression under the *T7lac* promoter on a second plasmid (the production plasmid). In this way, the GM‐CSF can be overexpressed (*T7lac* promoter), while the CheZ is expressed at natural levels (*hmp* promoter). Further, we have developed a range of relay plasmids that result in varying levels of expression via the production plasmid.

**Figure 4:**
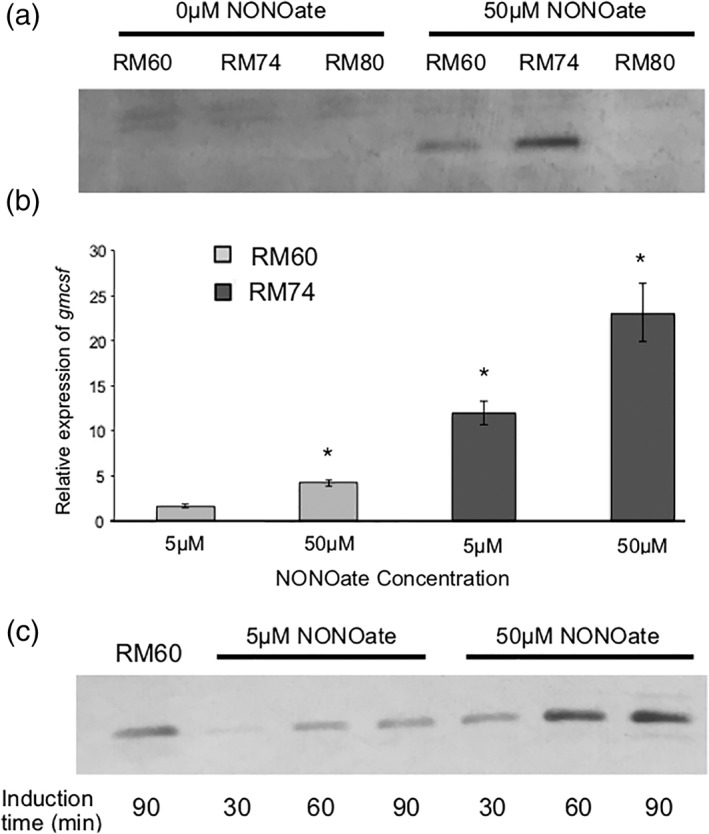
(a) Western blot against His‐tagged GM‐CSF. Cells are grown for 90 min with or without induction. Equivalent total protein is added per well as determined via the BCA assay, (b) quantitative PCR on gmcsf mRNA, using 16srRNA as a housekeeping gene. Samples are performed in triplicate and normalized as a fold‐change against RM60 cells at the zero timepoint (time of induction). Error bars represent the expression levels within one standard deviation, (c) Western blot against His‐tagged GM‐CSF from RM60 or RM74 cells. RM60 cells are induced with 50μM NONOate. Equivalent total protein is added per well as determined via the BCA assay. * Indicates *p* < .05 by ANOVA; all samples are statistically significant compared to the RM60 5μM baseline

That is, to investigate the ability of this system to express GM‐CSF, we created the pRM101 production plasmid, which expresses *gmcsf* under the *T7lac* promoter. From the selection of relay plasmids, we chose pRM44 and pRM52, which created RM74 and RM80 cells, respectively. To compare the ability of these cells to produce GM‐CSF versus cells that produce GM‐CSF under the *hmp* promoter alone (RM60), we performed qPCR and Western Blot analyses (Figure 4). Interestingly, RM80 cells were unable to produce GM‐CSF (Figure 4a). We believe this is attributed to a “fatal” combination of high expression seen in pRM52‐based systems, where the presence of the *ompA* tag on GM‐CSF resulted in destructive clogging of the secretion pathway.^37^, ^87^, ^88^, ^89^ Together, we believe that expression of this gene has to be tightly regulated in order to maintain a stable metabolic burden of the host bacteria. Despite this, both RM60 and RM74 cells could stably produce GM‐CSF upon induction, and exhibit no detectable protein without the presence of NO. Figure 4b corroborates the ability of RM74 cells to express higher levels of mRNA (and consequently protein in Figure 4a) than RM60 cells, confirming that the amplification circuit does indeed outperform the *hmp* promoter alone. Figure 4c illustrates that with varying levels of inducer, RM74 cells expectedly exhibit different levels of GM‐CSF production. While qualitative, the Western Blot indicates that RM60 cells with 50 μM NONOate produce roughly similar GM‐CSF levels as RM74 cells with only 5 μM NONOate after 90 min of induction. In summary, cells with the pRM44 relay plasmid were able to amplify a signal from NO and thus overproduce GM‐CSF with no observable complications (i.e., cessation of growth or aberrant morphology).

In order to release GM‐CSF from the periplasm, the construction of a pore in the outer membrane was our chosen approach, as discussed earlier. To overproduce the pore‐forming TolAIII protein, the gene was placed polycistronically under the *T7lac* promoter to create production plasmid pRM102. Thus, upon induction of cells with the pRM44 relay plasmid and pRM102 (RM75), elevated expression of GM‐CSF and TolAIII should occur. Additionally, toward our goal of achieving pseudotaxis, we generated a modification of pRM44 to include *cheZ‐YbaQ* expressed polycistronically after the *T7Pol* gene, here called pRM45. Notably, cells harboring pRM45 are a Δ*cheZ* host, while those with pRM44 are a wild‐type host. Cells RM74 and RM75 contain pRM44 and pRM101 or pRM102, respectively. Cells RM84 and RM85 are the Δ*cheZ* counterparts with pRM45 in place of pRM44. An illustration of RM85 is shown in Figure 7a.

**Figure 5:**
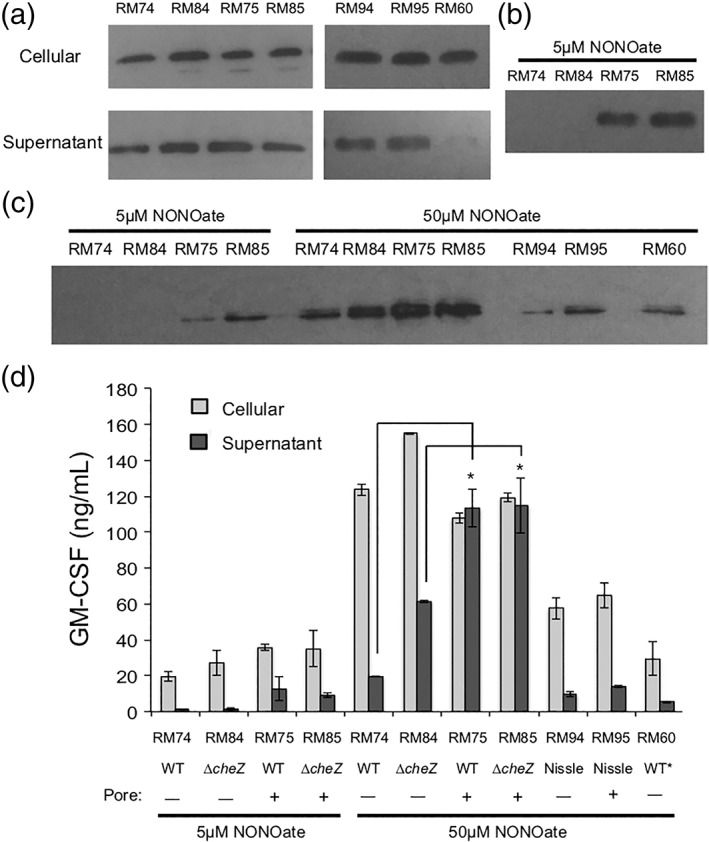
(a) Western blot against His‐tagged GM‐CSF. Cells are induced with 50μM DPTA/NONOate. Cellular fractions taken after 90 min of induction. Equivalent total protein as determined via BCA assay is loaded per well. Supernatants are sampled after 3 hr of induction and concentrated 33.3‐fold, (b) Western blot against His‐tagged GM‐CSF on supernatants of cells treated with 5 μM DPTA/NONOate for 3 hr; samples are concentrated 33.3‐fold, (c) Western blot against GroEL. Supernatants are sampled after 3 hr of induction and concentrated 33.3‐fold, (d) ELISA data using a competitive ELISA kit against His‐tagged GM‐CSF. Samples are performed in duplicate, and diluted to be within the detection range of the kit. Error bars are standard error. * Indicates *p* < .05 by ANOVA, suggesting that the pore‐less cells secrete statistically significant less GM‐CSF than their counterparts

Keeping in mind our goal of a probiotic host for an engineered system of GM‐CSF delivery, we implemented the pRM44 relay plasmid into *E. coli* Nissle 1917 cells, a commensal strain already used as a therapy for patients with IBD.^70^, ^71^, ^90^ The production plasmids pRM101 and pRM102 were also transformed, generating RM94 and RM95 cells, respectively. The capability of the aforementioned W3110 cells along with the Nissle strains to produce and secrete GM‐CSF into the extracellular space is presented in Figure 5; GM‐CSF was indeed produced in all examined cells, regardless of host strain or the presence of (a) the TolAIII pore, (b) the T7‐mediated amplification, or (c) expression of *cheZ* (Figure 5a). A blot on the supernatants of these bacteria reveals that cells harboring a relay plasmid for amplification contained GM‐CSF in their supernatants regardless of the presence of a TolAIII pore. Further, Figure 5b shows that in the supernatants of cells induced with the lesser 5 μM NONOate contained GM‐CSF exclusively in *tolAIII* ^+^ cells: those that contain the outer membrane pore. However, the presence of cytoplasmic GroEL in the supernatants indicates a significant leakage or lysis was occurring (Figure 5c). Notably, RM60 cells, which produce the least amount of GM‐CSF (Figure 4), do not show any detectable GM‐CSF in the supernatant. Supporting Information Figure S2c demonstrates that the lysis or leakiness of cells was not caused by high NO levels (50 μM NONOate), the cell density after the growth period, or the overexpression of protein (eGFP) via the *T7lac* promoter. Further, a growth curve of cells (Supporting Information Figure S3) reveals that overexpression of GM‐CSF ± TolAIII leads to an expected decrease in growth rate, but the small decline does not suggest significant cell lysis, indicating leakage as the likely primary mechanism of GroEL loss. Such phenomena of recombinant proteins promoting cell leakage have been reported, albeit without a defined mechanism.^91^, ^92^ Similarly, leakage of periplasmic proteins is often observed, although authors generally do not probe for cytoplasmic leakage as we have done here.^61^, ^89^, ^93^, ^94^ Concerning the expression system itself, no leaky expression of the *T7lac* promoter was detected via Western Blot (Supporting Information Figure S2d). Considering these findings, we believe the primary cause of GM‐CSF leakage in *tolAIII* ^−^ cells is a consequence of high levels of periplasmic GM‐CSF. Further discussion of observed leakiness can be found in the Supporting Information.

We next sought to quantify the levels of protein produced by the cells via ELISA (Figure 5d) to complement the qualitative analysis in Figure 5a–c. Cells induced with 5 μM NONOate saw elevated GM‐CSF levels in the supernatant, exclusively in samples with the TolAIII pore (RM75 and RM85). Similarly, supernatants of these cells when induced with 50 μM NONOate also contained more GM‐CSF than RM74 and RM84, confirming the function of the pore and a dose‐dependent response. As in Figure 5a,b, the host genome (WT vs. Δ*cheZ*) and subsequent NO‐induced expression of *cheZ* did not dramatically alter the levels of GM‐CSF produced. It was also discerned that despite the effects of the pore, significant amounts of GM‐CSF were yet intracellular. In sum, Figure 5 demonstrates (a) the utility of the TolAIII pore to facilitate periplasmic protein secretion, and (b) that the amplification circuitry indeed yields greater GM‐CSF (RM60 vs. all other cells).

### 3.3 Secreted GM‐CSF is biologically active

**Figure 6:**
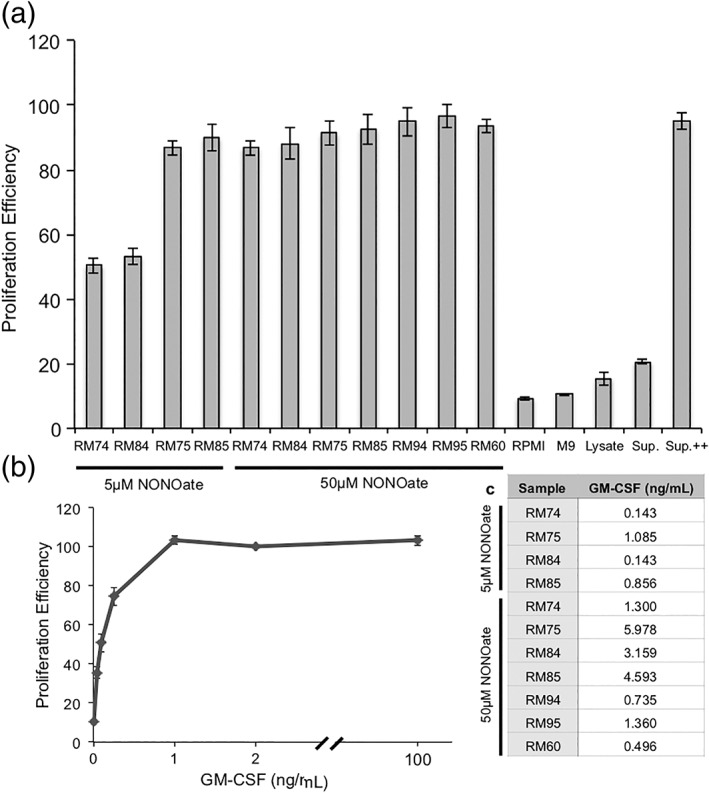
(a) Proliferation assay using TF‐1 erythroblasts, treated with an equal volume of controls or factors for approximately 96 hr. Baseline proliferation is determined by the fluorescence of cells treated with 2 ng/ml GM‐CSF after 1 hr of incubation with Alamar Blue. All data points are calculated as a percentage of this fluorescence in at least quadruplicate. Lysate refers to a lysate of ΔcheZ cells, and Sup. refers to a supernatant of uninduced RM74 cells, prepared in the same fashion as all induced cells. Sup. ++ is Sup. with 2 ng/ml GM‐CSF. Error bars are standard error, (b) proliferation assay providing a dose–response curve for varying levels of GM‐CSF. Baseline proliferation is determined by the fluorescence of cells treated with 2 ng/ml GM‐CSF after 1 hr of incubation with Alamar Blue. All data points are calculated as a percentage of this fluorescence in at least quadruplicate. Error bars are standard error, (c) approximate concentrations of GM‐CSF used in (a), calculated from the raw ELISA data from Figure 5d

Previous reports indicate that recombinant GM‐CSF produced in *E. coli* retains its biologic activity, despite lacking post‐translational modifications such as N‐ and O‐linked glycosylation.^49^, ^52^ With the addition of the *ompA* signal sequence and a C‐terminal histidine tag on GM‐CSF in this work, it was necessary to confirm that the protein was active. The cell line TF‐1 is completely dependent upon GM‐CSF for growth and thus serves as the standard method for assessing its activity via a proliferation assay. Briefly, cells were cultured with commercial GM‐CSF and resuspended in cytokine‐free media for the experiment, at which point they were supplemented with our produced GM‐CSF. Figure 6 demonstrates that GM‐CSF is indeed active in the supernatants from induced cultures. The diminished response observed using supernatants from RM74 and RM84 cells induced with only 5 μM NONOate is expected based on the concentrations of protein when compared to the dose–response curve; approximately 0.143 ng/ml GM‐CSF in these samples (Figure 6c) corresponds to reduced proliferation (Figure 6b). Due to the high amount of GM‐CSF produced in each sample, using 50 μl of supernatant results in similar proliferation levels among all samples (Supporting Information Figure S4). Additionally, the supernatants underwent at least three freeze–thaw cycles during the course of these experiments, suggesting a modest level of stability of the recombinant protein.

### 3.4 Combining pseudotaxis capabilities and GM‐CSF secretion

**Figure 7:**
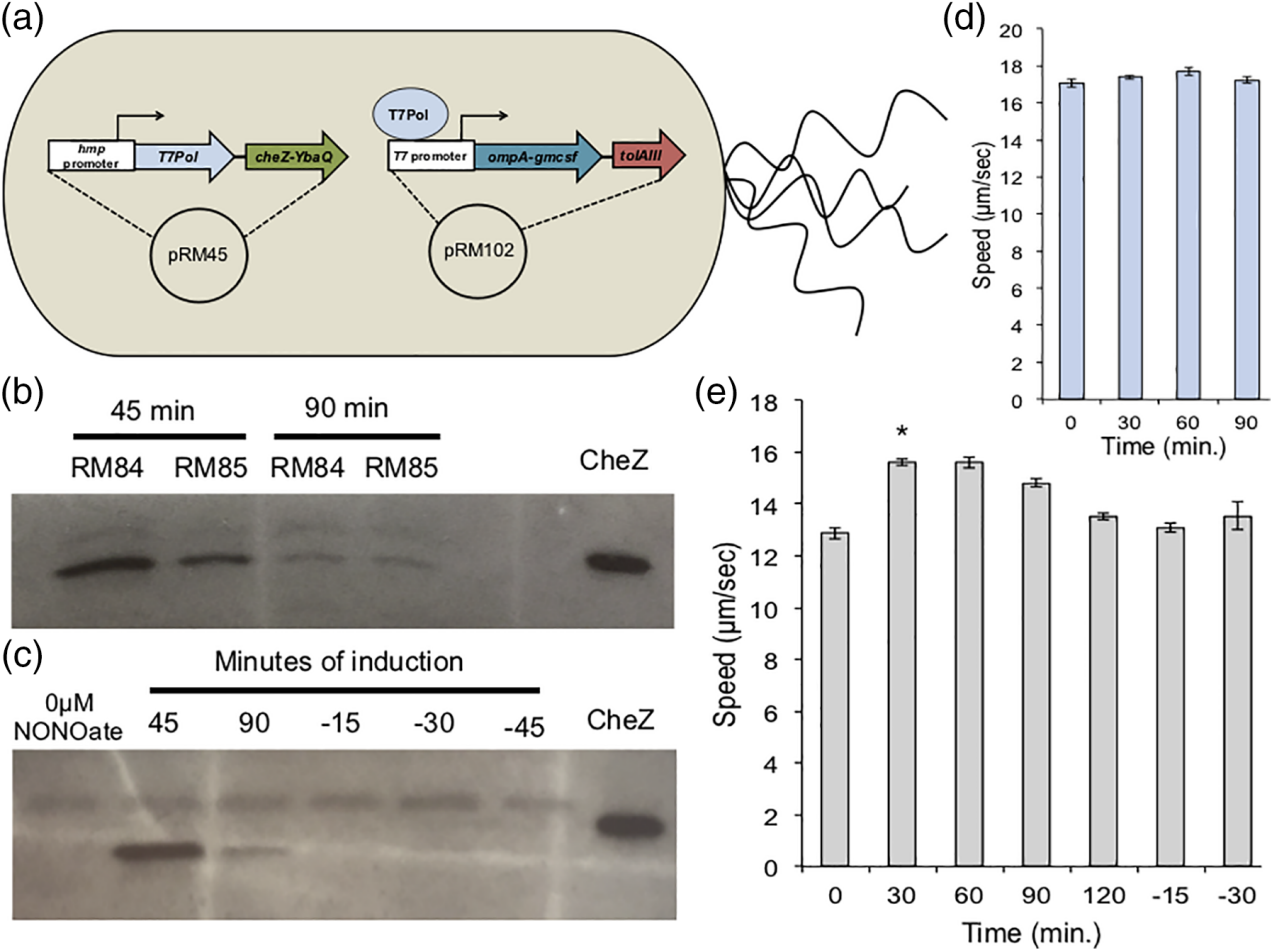
(a) Schematic of an RM85 cell: a Δ*cheZ* host bacterium with plasmids pRM45 and pRM102. (b, c) Western blot against CheZ. Cells are grown for 90 min with 50μM DPTA/NONOate. Only RM85 cells are observed in (c). Negative time values indicate samples resuspended in NONOate‐free growth media after 90 min of induction. Equivalent total protein is added per well as determined via the BCA assay, (d) speeds of RM75 (WT) cells over a time course induced with 50pM DPTA/NONOate. Error bars are standard error, (e) speeds of RM85 cells over a time course induced with 50μM DPTA/NONOate. Negative time values indicate samples resuspended in NONOate‐free growth media after 90 min of induction. Error bars are standard error. * Indicates *p* < .05 by ANOVA, to compare the induced sample to its un‐induced counterpart

In order to achieve a singular cell to serve as a delivery vehicle for GM‐CSF with a targeting mechanism, we incorporated both genetic circuits into one host cell. As discussed previously, Δ*cheZ* host cells are preferred for pseudotaxis, and thus these *E. coli* are transformed with plasmids pRM45 (expressing *T7Pol* and *cheZ‐YbaQ*) and pRM101 or pRM102 (expressing *gmcsf* ± *tolAIII*) to produce RM84 and RM85 cells, respectively. Figure 7b,c presents immunoblots which show that upon induction, RM84 and RM85 cells produced similar levels of CheZ, indicating that the expression of *tolAIII* has little or no effect on *cheZ* expression. Further, the degradation of CheZ via the YbaQ tag depletes CheZ levels even during the 90 min induction timecourse. RM24 cells, lacking the GM‐CSF production and secretion mechanism, did not experience this rapid loss of CheZ during the timecourse (Figure 2b), suggesting that overproduction of GM‐CSF reduces the level of *cheZ* expression. Despite the lesser values of CheZ produced in RM84 and RM85 cells versus those presented in Figure 2, the levels of CheZ present in the induced cells were indeed able to confer a change in motility. Figure 7e reveals that RM85 cells, once induced, quickly regain the ability to “run” and their swimming speed increases to approach values found among *cheZ* ^+^ RM75 cells in Figure 7d. Even without changing the growth media to remove NONOate, cells reverted back to a tumbling phenotype after 2 hr of incubation with NO, suggesting that the YbaQ tag sufficiently depleted CheZ levels in the cells. As discussed above, NO has likely been fully consumed by this point, and thus expression of *cheZ* has minimized such that the degradation outperforms the accumulation within this timeframe. Unsurprisingly, resuspending the cells in NONOate‐free media after 90 min of induction also resulted in tumbling cells (Figure 7e, −15 and −30 timepoints). As noted previously, a rapid loss of motility is beneficial for pseudotaxis purposes, as it encourages the engineered cells to slow down when exiting pseudoattractant (i.e., NO) rich areas.^21^ The trends observed in Figures 2 and 7 where RM24 and RM85 cells, respectively, have their motility restored in the presence of nitric oxide but quickly become tumbly in the absence of the inducer, mirror the behavior of pseudotactic cells previously reported.^21^ As discussed earlier, we therefore believe by extension that the Δ*cheZ* cells engineered in this study to produce CheZ‐YbaQ in response to NO should exhibit pseudotaxis and accumulate near sources of nitric oxide. Their ultimate ability to be retained near NO‐rich locales naturally will depend on many factors, however.

### 3.5 Using an *in vitro* Crohn's disease model to stimulate GM‐CSF production

Using the pseudotaxis‐capable delivery vehicle cells RM85, and the probiotic proof‐of‐concept RM95, we sought to confirm their response to a simple CD in vitro model consisting of inflamed intestinal epithelial cells using the Caco‐2 cell line. Briefly, a confluent layer of Caco‐2 cells secrete a basal level of NO, and we stimulate a sub‐population with pro‐inflammatory cytokines to significantly increase their NO production to mimic inflammation.^95^, ^96^, ^97^ We then exposed our engineered bacteria to the supernatant of the Caco‐2 cells (i.e., the “lumen”) for 90 min, and analyzed their *gmcsf* expression (Supporting Information Figure S5). The results indicate a small increase in *gmcsf* expression in response to the basal NO secretion, and, importantly an elevated response (up to 20‐fold) when exposed to the inflamed model. We estimate that the NO level in these cells may be upwards of 20 μM.^97^ Ultimately, this confirms the activation of our bacteria in response to biologically produced NO with a relevant model using intestinal epithelial cells. Further studies should include the use of HT29‐MTX cells in co‐culture with Caco‐2, however, to better mimic the native intestinal lumen; the model would include the mucus barrier, which may interfere with the NO release kinetics and thus GM‐CSF secretion.^98^

## 4 CONCLUSIONS

We have developed probiotic bacteria that overproduce and secrete a biologically active human therapeutic, GM‐CSF, in the presence of the biomarker nitric oxide. Additionally, we successfully demonstrated a pseudotactic motility circuit also regulated by NO. Ultimately, we envision these concepts and the design methodologies developed here may be combined with in vivo models of IBD diseases such as CD, to generate therapeutic probiotics with enhanced targeting abilities. Such a treatment modality may diminish side effects and improve convenience versus current intravenous and therefore systemic deliveries; more broadly, via cloning of different therapeutic genes it serves as a modular platform to treat other gastrointestinal ailments.

## Supporting information

Video S1

Video S2

Video S3

Video S4

Appendix S1: Supinfo
