# Supplementary material for: A platform of genetically engineered bacteria as vehicles for localized delivery of therapeutics: Toward applications for Crohn's disease
Source: Bioeng Transl Med. 2018 Sep 23;3(3):209–21. doi: 10.1002/btm2.10113 (PMC6195910; doi:10.1002/btm2.10113)
Supplement: Supplementary file 5 — Appendix S1: Supinfo [file BTM2-3-209-s005.pdf]

Supplemental Information

Methods

Plasmid Construction. pRM11 and pRM24: As described previously for plasmid pRM01, except the *cheZ* (from pHW01) or *cheZ-YbaQ* (from pWM1) gene was amplified instead of *egfp*.[1] pRM31-pRM32: These plasmids were created nearly identically to pRM11 and pRM24. The SpeI and SacI sites are used in pGEM-T-Easy rather than SpeI and SalI. The *cheY* gene was amplified from the WT genome and *cheY*^{13DK106YW} (*cheY***) from pXYZ202. pRM42 and pRM44: Described previously.[1] pRM45: A pST39 derivative containing the *hmp* promoter with *T7Pol* including 6 extra nucleotides prior to the start codon and lacking the BamHI site (described previously)[1] is digested with EcoRI and HindIII and a *cheZ-YbaQ* gene amplified with EcoRI and HindIII sites is inserted here. A positive clone is used as a template for PCR to amplify the insert with BamHI and XhoI sites, and is subsequently digested and inserted into pBSh. pRM60: The *ompA-gmcsf-c-myc*-his6 gene is amplified from pGM29ompA using a set of primers designed with the New England Biolabs (NEB) web-tool for Gibson Assembly. A pBSh derivative with the *hmp* promoter is similarly amplified with the Gibson Assembly primers. The pBSh and *ompA-gmcsf-c-myc*-his6 PCR products are ligated following NEB's protocol. pRM101: The *ompA-gmcsf-c-myc*-his6 gene is amplified from pGM29ompA with NdeI and SacI sites and inserted into pET200. pRM102: The *ompA-gmcsf-c-myc*-his6 gene is amplified from pGM29ompA using a set of primers designed with the NEB web-tool for Gibson Assembly. pST39 is similarly amplified with the Gibson Assembly primers. The pST39 and *ompA-gmcsf-c-myc*-his6 PCR products are ligated following NEB's protocol to insert the gene after the XbaI site. The *rbp-tolAIII* gene is amplified from pTA3Y with EcoRI and HindIII sites, and is digested and inserted into the aforementioned pST39 derivative. A positive clone is used as a template for PCR to amplify the insert with NdeI and SacI sites, and is subsequently digested and inserted into pET200.

Results & Discussion

A brief discussion of Supplemental Figures is within their caption.

Supplemental Videos. The following videos have been uploaded with submission:

1. RM15 uninduced.avi
2. RM15 induced.avi
3. RM16 uninduced.avi
4. RM16 induced.avi

These videos illustrate that Δ*hmp* host cells have similar motility characteristics to wild-type cells, however in the presence of nitric oxide, Δ*hmp* host cells lose all motility functionality. Further, Δ*hmp*Δ*cheZ* host cells are tumbly by default due to lack of CheZ, however also lose all motility functionality when challenged with NO.

Observed Leakiness in GM-CSF Overproducing Cells.

Notably, only a small amount of GroEL is detected in the supernatant of *tolAIII*$^+$ cells induced with 5μM (Figure S2b), and thus it is possible that the outer membrane pore contributes to leakiness of cells in addition to overproduced GM-CSF. However, a previous report indicates that the TolAIII pore should not compromise the integrity of the inner membrane of cells.[2] Thus, we believe the leakiness is due to a combination of shuttling both ompA-GM-CSF and TolAIII proteins across the inner membrane, such that the total amount of cross-membrane shuttling results in damage that facilitates leakage of proteins. RM74 and RM84 cells lacking *tolAIII* but induced with 50μM NONOate are producing an abundance of GM-CSF, of which the continuous burden of the single translocating protein may be sufficient to cause membrane damage. In summary, the total amount of protein to be shuttled across the inner membrane we believe is likely responsible for cell leakiness in this study.

Despite this observed leakiness of cytoplasmic components, we note that the concern is likely minimal. Commensal bacteria undergo death and lysis within the body and consequently release intracellular components continuously with no deleterious effects on the host organism (i.e. human). Rather, with these engineered cells, the leakiness may be of benefit as it releases recombinant GM-CSF that has remained within the cell, as only a maximum amount is able to be shuttled through the inner membrane.

References

(1) McKay, R., Hauk, P., Quan, D., and Bentley, W. E. (2018) Development of Cell-Based Sentinels for Nitric Oxide: Ensuring Marker Expression and Unimodality. *ACS Synth. Biol.* Submitted.

(2) Wan, E. W., and Baneyx, F. (1998) TolAIII co-overexpression facilitates the recovery of periplasmic recombinant proteins into the growth medium of *Escherichia coli*. *Protein Expr. Purif. 14*, 13–22.

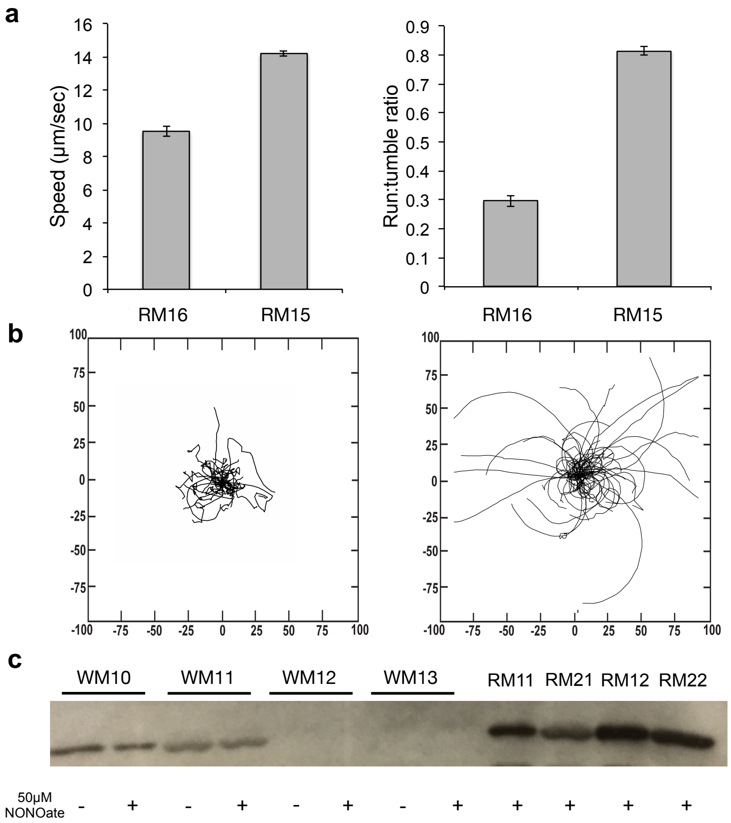

Figure S1. Hmp has no inherent effect on motility. (a) Speed and run:tumble ratio of Δ*hmp*Δ*chez* RM16 cells and Δ*hmp* RM15 cells determined using TumbleScore. Error bars are standard error. Cells are grown to log phase and resuspended in chemotaxis buffer. (b) Accompanying rose graphs of RM16 cells (left) and RM15 cells (right). Distance on axes is µm. Δ*hmp* cells expectedly swim faster and "run" more than Δ*cheZ*Δ*hmp* cells, due to genomic CheZ production (c) Western blot against CheZ in cells induced for 90 minutes, or grown without induction. Equivalent total protein is loaded per well as determined by the BCA assay. The addition of plasmid pT5G does not significantly reduce the level of CheZ produced (WM11 vs WM10, RM21 vs RM11, RM22 vs RM12). Further, cells lacking *hmp* (RM12 and RM22 vs. RM11 and RM21) produce slightly elevated levels of CheZ due to greater overall NO exposure.

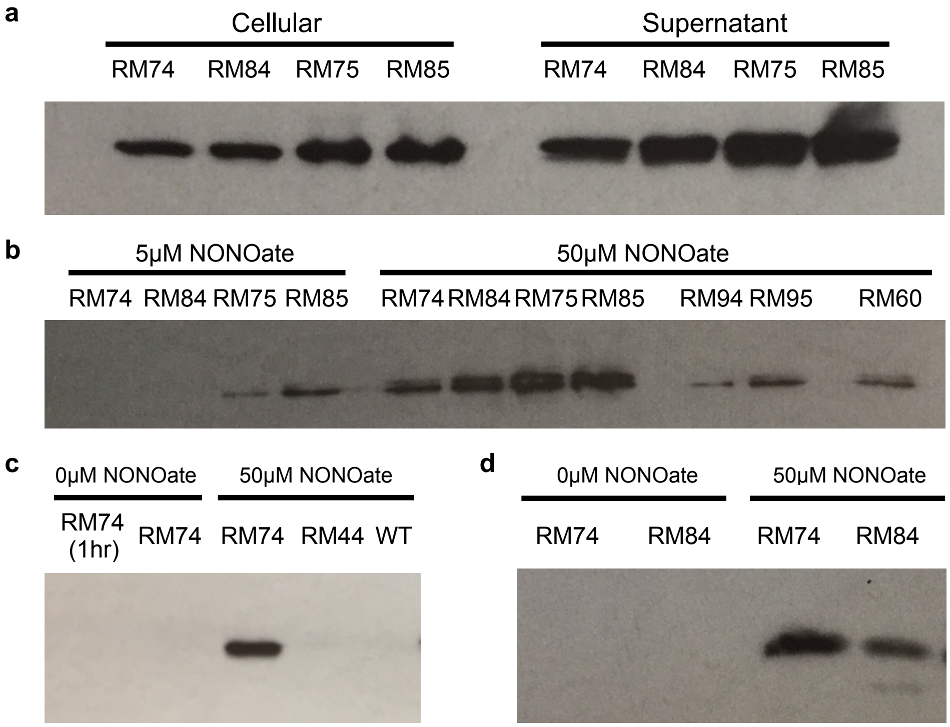

Figure S2. Cytoplasmic leakage is due to membrane-translocating protein overproduction. (a-c) Western Blot against GroEL using cells induced for 90 minutes with 50μM NONOate or without induction. Samples in (b-c) are exclusively supernatants. Blot in (a) confirms both intracellular and extracellular GroEL. In (c), RM74 cells induced for 1 versus 3 hours reveals that the increase in cell density does not contribute to GroEL leakage. Induction of RM44 cells which overproduce eGFP via the *T7lac* amplification circuit does not contribute to GroEL leakage. The addition of NO to cells does not contribute to GroEL leakage, indicated by the WT sample. (d) Western Blot of supernatants against His-tagged GM-CSF using cells induced for 90 minutes with 50μM NONOate or without induction demonstrates no detectable leaky expression. Equivalent total protein is loaded per well for each blot, determined via BCA assay.

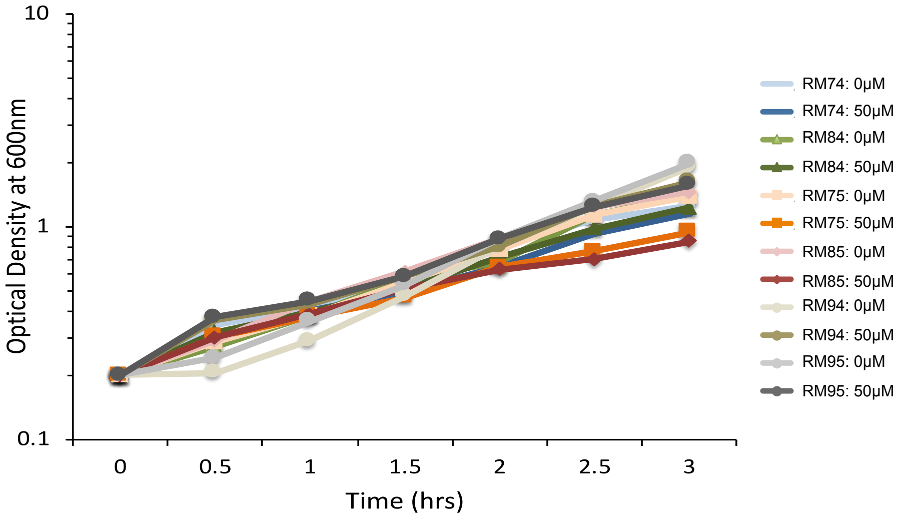

Figure S3. Cumulative protein production has a minimal effect on growth rate. Growth curve of cells untreated or induced with 50μM DPTA/NONOate. Data points begin at an OD_{600} of 0.2. Cells expressing the most proteins (induced RM85 produce CheZ-YbaQ, T7Pol, GM-CSF, and TolAIII) expectedly have the slowest growth

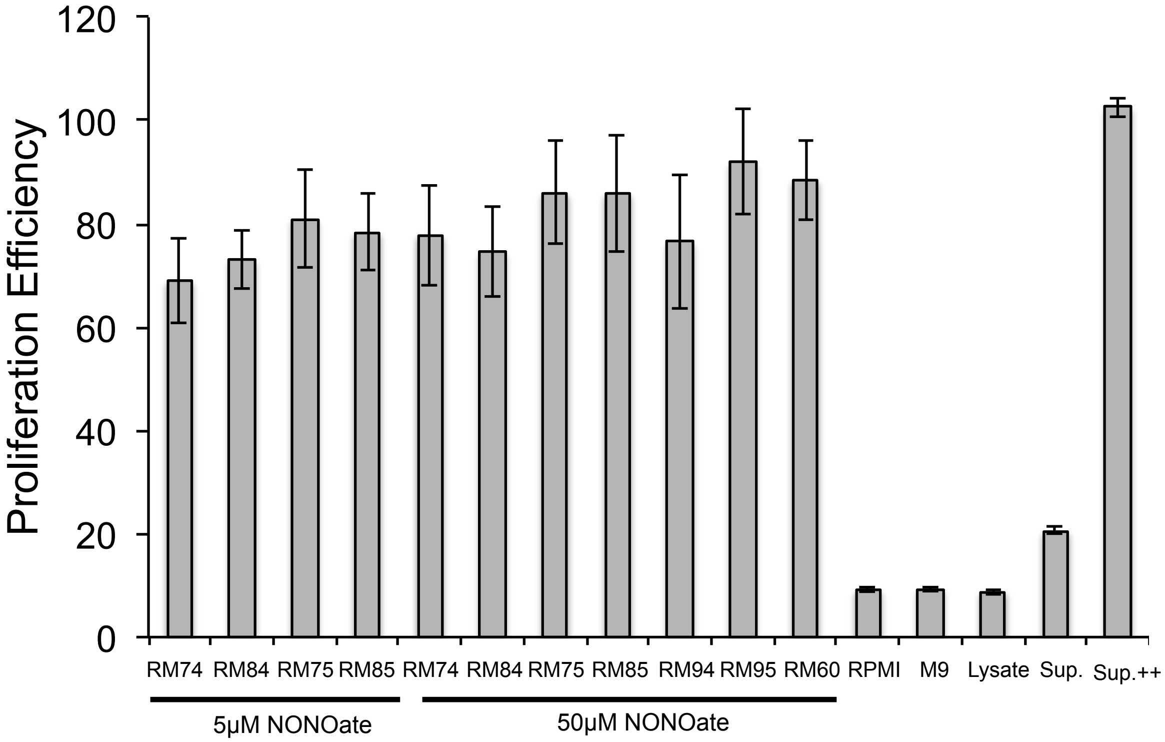

Figure S4. Addition of surplus GM-CSF yields a higher proliferation response. Proliferation assay using TF-1 erythroblasts, treated with an equal volume of controls or factors for approximately 96 hours; 10 times more cell lysate is added per sample than in Fig. 6a. Baseline proliferation is determined by the fluorescence of cells treated with 2 ng/mL GM-CSF after 1 hour of incubation with Alamar Blue. All data points are calculated as a percentage of this fluorescence in at least quadruplicate. Lysate refers to a lysate of Δ*cheZ* cells, and Sup. refers to a supernatant of uninduced RM74 cells, prepared in the same fashion as all induced cells. Sup. ++ is Sup. with 2ng/ mL GM-CSF. Error bars are standard error.

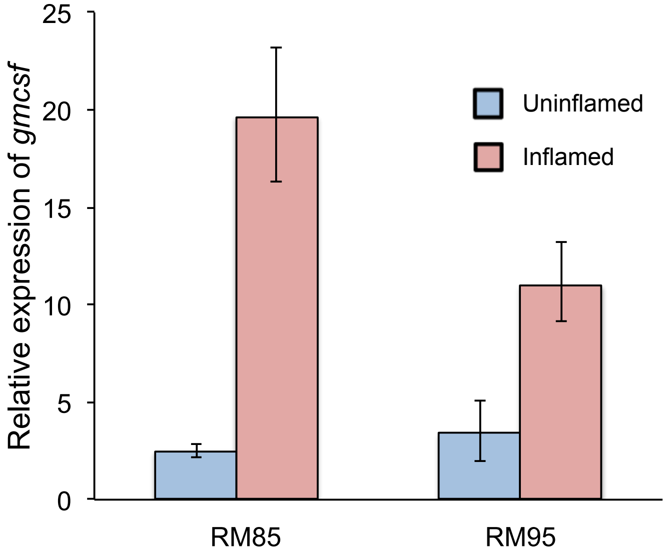

Figure S5. Biologically produced nitric oxide stimulates *gmcsf* expression. Quantitative PCR on *gmcsf* mRNA, using *16srRNA* as an endogenous housekeeping gene. Samples are performed in triplicate and normalized as a fold-change against RM85 cells grown to log phase in a tube (not exposed to Caco-2 cells). Bacteria are exposed to Caco-2 cultures for 90 minutes prior to RNA isolation. Error bars represent the expression levels within one standard deviation.
